# Characterisation and validation of Mel38; A multi-tissue microRNA signature of cutaneous melanoma

**DOI:** 10.1371/journal.pone.0211504

**Published:** 2019-02-05

**Authors:** Ryan Van Laar, Mitchel Lincoln, Sian Fereday

**Affiliations:** Geneseq Biosciences, Melbourne, Australia; University of South Alabama Mitchell Cancer Institute, UNITED STATES

## Abstract

**Background:**

Histopathologic examination of melanocytic neoplasms can be challenging and subjective, with no specific circulating or tissue-based biomarkers currently available. Recently, a circulating 38-microRNA profile of melanoma (Mel38) was described. In this study, Mel38 expression and its impact on downstream mRNA regulation in solid tissue is examined.

**Methods:**

Mel38 was applied to archival, clinically-annotated, solid-tissue genomic datasets representing benign naevi, primary and metastatic melanoma. Statistical analysis of the signature in relation to disease status, patient outcome and molecular pathways was performed.

**Results:**

Mel38 is able to stratify genomic data from solid tissue biopsies on the basis of disease status and differences in melanoma-specific survival. Experimentally-verified messenger-RNA targets of Mel38 also exhibit prognostic expression patterns and represent key molecular pathways and events in melanoma development and progression.

**Conclusion:**

The Mel38 microRNA profile may have diagnostic and prognostic utility in solid tissue as well as being a robust circulating biomarker of melanoma.

## Introduction

The worldwide incidence of melanoma skin cancer continues to increase, outpacing the rise of any other malignancy in the white population over the last 30 years [1, 2]. In 2015 alone, approximately 351,880 melanoma skin cancers were diagnosed [3]. The diagnosis of cutaneous melanocytic lesions relies on a pathologist’s visual assessment of biopsy material on microscopic slides, a process which multiple studies have shown to have low accuracy and reproducibility [4–6]. The study by Elmore et al revealed that up to 1 in 6 melanomas may be misdiagnosed [4].

Estimates for numbers of pigmented lesions (seborrhoeic keratoses, naevi and melanoma) excised for each malignant melanoma identified range widely, from 6.3 [7] to 19.6 [8]. At the current global incidence of melanoma, this equates to between 2.4 million and 6.9 million skin lesion histopathology procedures being performed each year in response to a clinical suspicion of melanoma. These occur at a significant cost to global healthcare systems and potential patient morbidity associated with the biopsy procedure, including the risk of biopsy infection and disfiguration [9, 10].

Molecular markers of melanoma that can be performed on solid and/or “liquid biopsies” (eg. blood or urine) have been proposed as one approach to improve diagnostic accuracy and reduce the rate of both over and under diagnosis [4]. MicroRNAs are key regulators and drivers of cellular events that lead to the development and progression of melanoma. Because disease-specific patterns of microRNA expression can be reliably detected in both solid tissue and biofluids including blood and urine, they have the potential to assist in the diagnosis and monitoring of melanoma at both a local (biopsy) and systemic (blood) level.

Mel38 (Melaseq) is a recently developed microRNA expression signature, identified by genomic profiling of plasma from individuals with or without cutaneous melanoma, including patients with stage IA—IV disease [11]. The 38-microRNA signature was validated on multiple independent datasets representing blood, cell-lines and solid tissue. Mel38 has additionally been demonstrated to have potential as a marker of post-excision treatment response [12].

In this study, we apply the Mel38 signature to multiple previously published genomic datasets to further examine its biological and clinical significance. Both microRNA and protein-coding, messenger-RNA (mRNA), datasets are included, to address the following questions:

1. Does Mel38 differentiate between solid tissue biopsies of benign naevi, primary and metastatic melanoma?
2. Does Mel38 predict patient outcome (i.e. melanoma-specific survival)?
3. What are the downstream molecular functions and pathways targeted/regulated by Mel38?
4. Are expression levels of the Mel38-regulated mRNAs also associated with melanoma-specific survival?

While other groups have described genomic signatures for melanoma diagnosis or prognosis, to our knowledge this is the first time a circulating microRNA signature has been applied to genomic profiles of solid tissue, which is routinely used in the diagnosis of melanoma. Additionally, the use of archival data generated in multiple laboratories, with a range genomic technologies and specimen preparation methods also provides an assessment of the algorithms robustness and its potential for improving the accuracy of melanoma diagnoses in clinical practice.

## Patients & methods

Details of the genomic datasets download from NCBI Gene Expression Omnibus (GEO) and used in this study are shown in Table 1 [13]. Patient demographics, specimen processing and genomic profiling methods are described in the respective original publications [14–16]. All data were downloaded in pre-normalised format and probe IDs were matched to the Mel38 signature by comparing probe annotations and sequence information, obtained from the respective manufacturer’s website.

**Table 1:** Description of previously-published genomic datasets used in this study.

NCBI GEO ID	Description	Number of unique samples	Molecule detected	Genomic platform
GSE35579	Formalin-fixed and paraffin-embedded samples from different stages of melanomagenesis [14].	52	microRNA	Melton lab-Human melanoma-71-v2-microRNA Illumina BeadChip
GSE59334	Fresh-frozen biopsies from stage III melanoma patients with outcome data [15].	74	microRNA	Agilent Human miRNA V16 oligonucleotide microarray
GSE65904	Whole-genome expression analysis of melanoma tumour biopsies from a population-based cohort [16].	214	mRNA	Illumina HumanHT-12 V4.0 expression beadchip

Multi-dimensional scaling of genomic data was performed using principal component analysis (PCA) to generate x/y/z axis values. Prognostic models were developed using the Cox proportional-hazards method was used to relate survival time to first 2 PCA linear combinations of microRNA expression levels using BRB Arraytools [17]. A regression coefficient (weight) for each principal component was used in conjunction with leave-one-out cross validation to compute a prognostic index for each patient. The prognostic index correlates with the hazard of death and can was used to assign patients to discrete risk groups based on a chosen threshold.

To analyse the relationship between genomic risk groups (microRNA and mRNA) and melanoma-specific survival, Kaplan Meier analysis with Log rank testing and cox proportional-hazards regression were performed. P-values of less than 0.05 were considered statistically significant. All statistical analyses and visualisation were performed using MedCalc v18.9 [18]. Molecular pathway and gene ontology analysis was performed using the Database for Annotation, Visualization and Integrated Discovery (DAVID) v6.8 [19].

## Results

### The Mel38 microRNA signature is able to stratify FFPE tissue biopsies of benign naevi, primary and metastatic melanoma

To explore the ability of the Melaseq Mel38 signature to function as biomarker to aid in the histological diagnosis of melanoma, dataset GSE35579 was downloaded from the NCBI Gene Expression Omnibus. This dataset was generated by the Melton Lab at the University of Edinburgh, using a customised version of the Illumina microRNA BeadChip (NCBI platform ID: GPL15183) [14]. As described in the original publication associated with these data, total RNA was isolated from 52 FFPE samples, comprising of 11 benign naevi, 20 primary melanoma and 21 metastatic melanoma biopsies.

**Fig 1:**
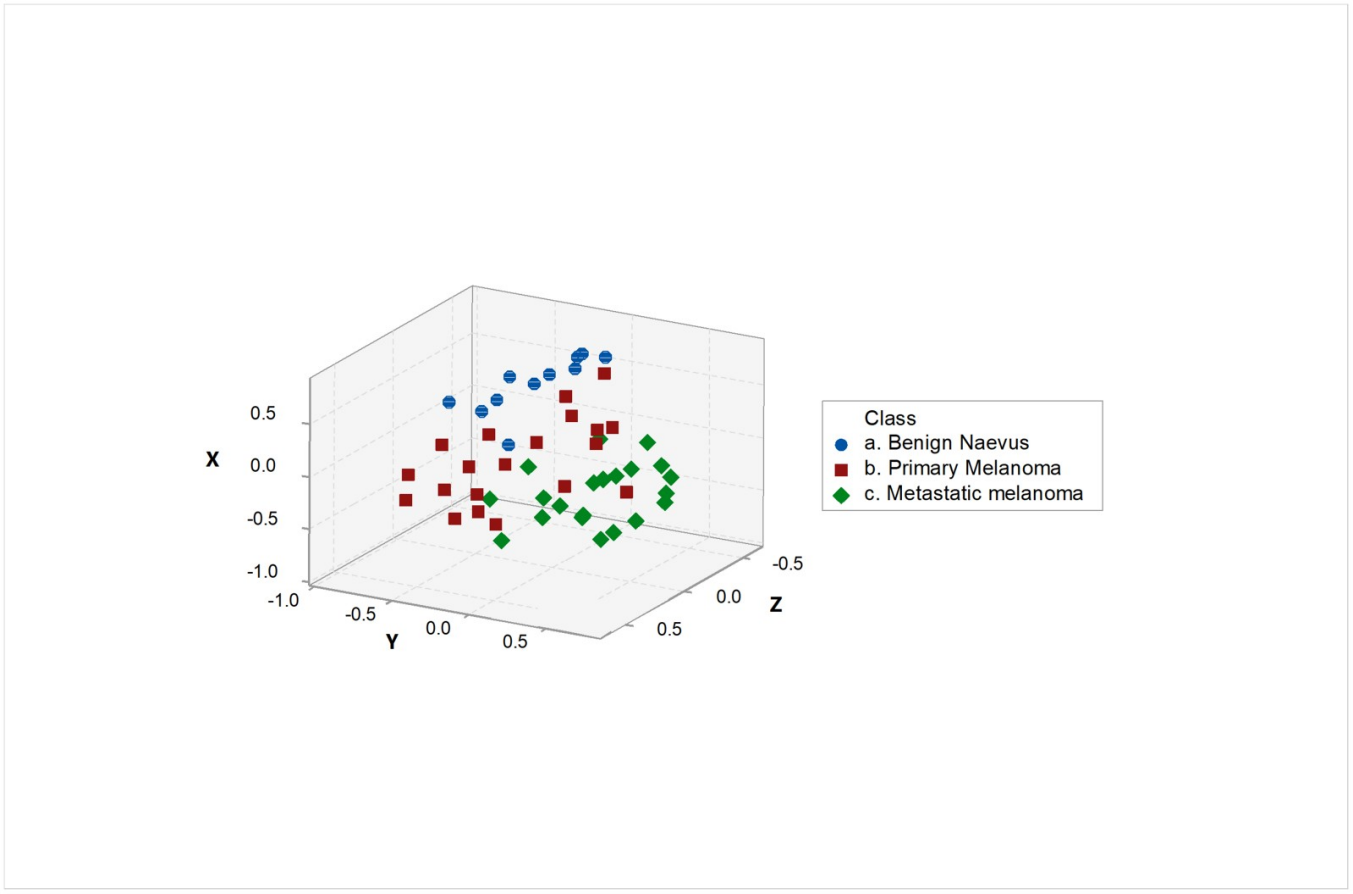
Multidimensional scaling of 52 FFPE samples of benign naevi, primary and metastatic melanoma based on their expression of a 27-microRNA subset of the Mel38 signature. X, Y and Z axis correspond to the first 3 principal components present in the 52 sample x 27 microRNA-expression dataset.

The microRNA analysis platform used contained 735 targets, representing 470 well-annotated human microRNAs. Target matching to Mel38 was performed using target annotation files and individual probe sequences. Twenty seven of the 38 microRNAs were identified from this process (71%). Multidimensional scaling of samples was performed using the 27 gene subset, using PCA to summarize the genomic data into three dimensions. As shown in Fig 1, clustering of samples represents the three disease stages of melanoma progression represented by this series; benign naevi, primary and metastatic melanoma.

To determine the ability of this majority-subset of Mel38 to discriminate between FFPE specimen types using the same type of classifier as previously described, a support vector machine (SVM) algorithm applied to the data using the binary classes of benign vs melanoma. The binary classification accuracy observed from this exercise was 94–96%, depending on the cross-validation method (leave one out, 10-fold or 0.632+bootstrap). Sensitivity and specificity for prediction of melanoma status was 0.97 and 0.85 respectively. The classifiers positive predictive value was 0.96 and its negative predictive value was 0.89.

**Fig 2:**
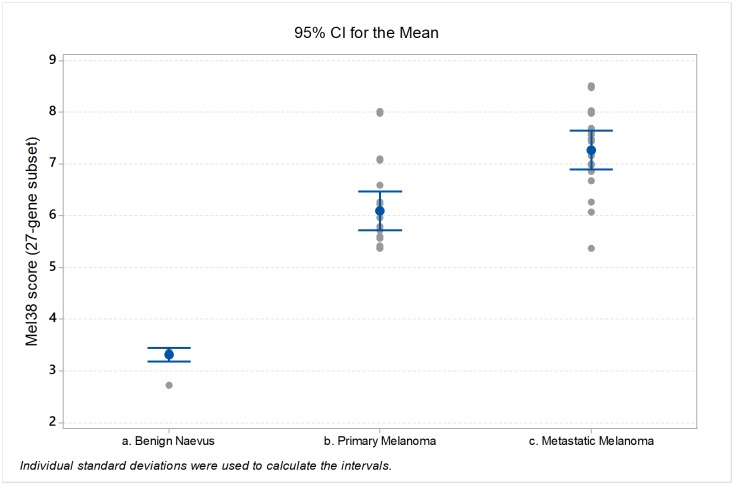
Individual value plots of FFPE-microRNA classification scores, grouped by disease status. Scores were generated using a support vector machine algorithm trained on the 27-microRNA subset of the Mel38 signature. Mean and 95% confidence intervals of each group shown.

To visualise the output of the SVM classifier, the trained model was applied to the 27-microRNA x 52 specimen dataset by calculating the sum of weighted microRNA abundance measurements. The resulting classification scores are shown in Fig 2. The mean classification score for the benign nevus class was 3.3, compared to 6.7 for the combined primary and metastatic melanoma class. Within the melanoma category, primary tumours had a mean classification score of 5.4, compared to 7.3 for the metastatic cases (T-test P = 2.03 x 10^−5^). These data further support the hypothesis that the Mel38 microRNA signature is able to differentiate between benign naevi and primary or metastatic melanoma.

### The Mel38 microRNA signature is predictive of melanoma specific survival

To explore the relationship between the Mel38 signature and patient outcome, dataset GSE59334 was downloaded from NCBI GEO. This dataset contains Agilent Human miRNA V16 oligonucleotide microarray profiles of fresh-frozen lymph node specimens obtained from 74 stage III (AJCC 5^th^ edition) melanoma patients.

**Fig 3:**
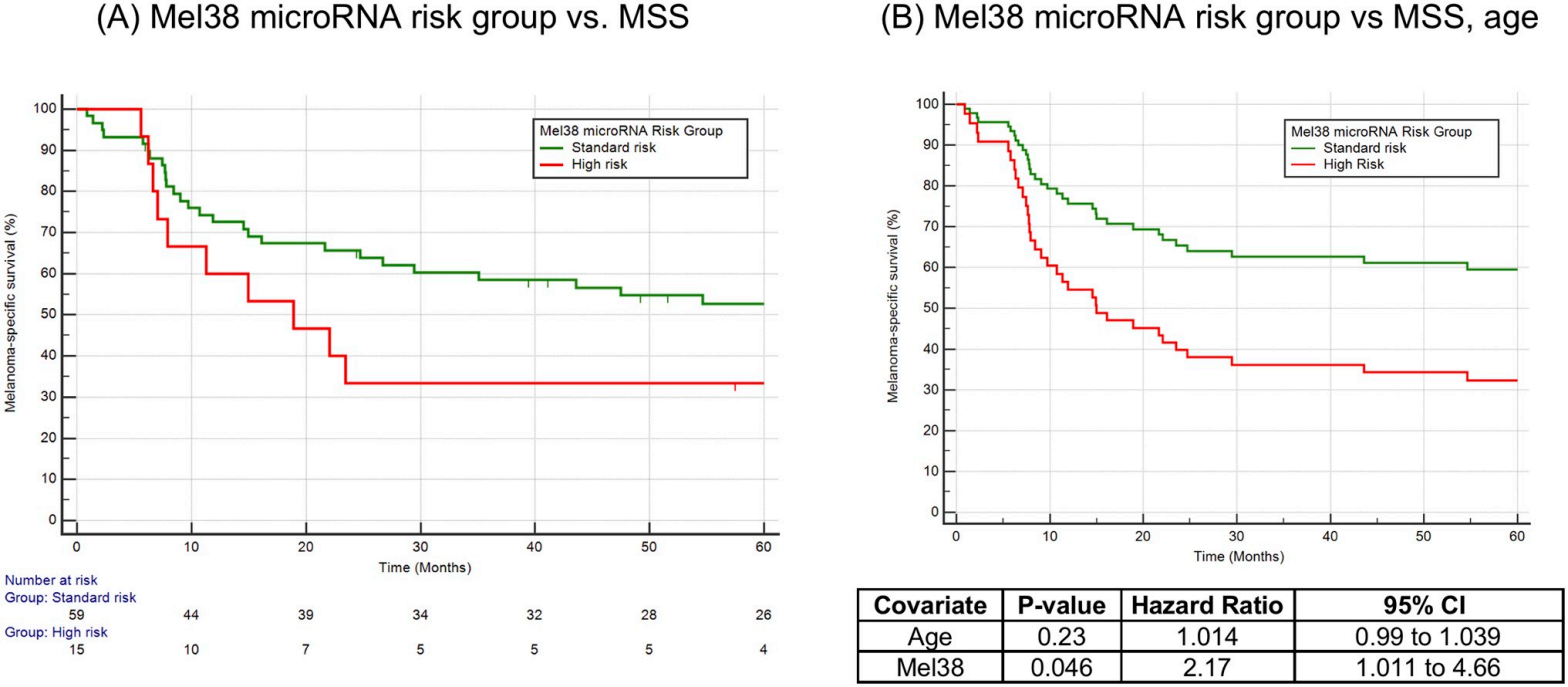
(A) Kaplan Meier analysis of stage III melanoma patients in the high-risk vs standard risk group, as defined by a cross validated prognostic model (Log rank P-value = 0.061) (B) Multivariate cox proportional hazards regression of microRNA risk group, adjusted for patient age at specimen collection. Mel38 significantly stratifies patients for 5-year MSS independent to age at diagnosis (P = 0.046).

Agilent microarray probes corresponding to the Mel38 microRNAs were identified using probe annotation files and sequence comparison. Thirty-three matches were found (87%) and were used generate a prognostic score for each patient using leave-one-out cross validation and the weighted principal component analysis method. Kaplan Meier analysis was performed for difference in 5-year melanoma-specific-survival between patients in the top quintile (‘high risk’, n = 15) versus the remaining 80% of the cohort (‘standard risk’ n = 59), as shown in Fig 3A. This resulted in a hazard ratio of 2.00 (95% CI 0.82 to 4.88) and Log rank test P-value that approached statistical significance (P = 0.061).

A multivariate cox regression analysis was also performed using the patients age at the time of the specimen collection and the Mel38 high/standard risk group (Fig 3B). When adjusted for patient age, the hazard ratio of the genomic risk group was 2.17 (95% CI: 1.011 to 4.66), P = 0.046. Regression analysis using the prognostic risk score as a continuous variable, rather than 80/20 standard/high risk groups, resulted in a hazard ratio of 2.63 (95% CI: 1.17 to 5.92), P = 0.020.

These data indicate that in a patient series limited to stage III patients only, a 32-microRNA subset of the Mel38 signature is able to stratify patients into high or standard risk groups with statistically significant differences in five-year MSS, when adjusted for age at the time of specimen collection.

### Messenger RNA’s regulated by Mel38 have key roles in melanoma development and progression

Using the miRTarBase (version 7.0) database of functional microRNA-mRNA interactions, 329 protein coding genes (messenger RNAs) were identified as the targets of the Mel38 microRNA signature [20]. To investigate the function of this Mel38-regulated gene set in melanoma, they were analysed in a clinically-annotated whole-genome mRNA dataset, generated from 214 melanoma tumour biopsies (NCBI GEO ID: GSE65904). Fifty-one out of 239 genes were excluded from further analysis due to undetectable expression or non-significant variance between patients (ie. median log2 intensity < 10, variance P > 0.01). This resulted in a set of 278 mRNAs with detectable expression in melanoma tissue and experimentally-verified functional interactions with the Mel38 microRNA signature.

#### Molecular pathway & gene ontology analysis

Molecular pathway and gene ontology enrichment was performed on the 278 Mel38 targets using the DAVID 6.8 Functional Annotation Tool with minimum gene set overlap of 5 genes and Benjamini adjusted p-value of <0.05. Relevant and significantly over-represented KEGG and Biocarta molecular pathways are shown in Tables 2 and 3, respectively. The results of both database analyses are dominated by signalling, cell cycle regulation and proliferation pathways strongly associated with melanoma development and progression, including PI3K-Akt and MAPK pathways[20].

**Table 2:** Significantly represented KEGG molecular pathways in the Mel38-regulated mRNA gene set.

KEGG Pathway	Count	Percent of gene set	P-Value	Fold Enrichment	Adjusted P-value (Benjamini)
hsa05206: MicroRNAs in cancer	45	16.19	1.60E-21	5.60	3.30E-19
hsa05200: Pathways in cancer	52	18.71	1.75E-21	4.69	1.81E-19
hsa04151: PI3K-Akt signaling pathway	41	14.75	4.97E-15	4.21	2.59E-13
hsa05218: Melanoma	19	6.83	4.51E-13	9.48	1.33E-11
hsa04066: HIF-1 signaling pathway	20	7.19	1.70E-11	7.23	3.52E-10
hsa04510: Focal adhesion	24	8.63	1.15E-08	4.13	1.40E-07
hsa04110: Cell cycle	18	6.47	5.14E-08	5.14	5.32E-07
hsa04012: ErbB signaling pathway	14	5.04	7.45E-07	5.70	5.71E-06
hsa04115: p53 signaling pathway	12	4.32	2.09E-06	6.35	1.49E-05
hsa04014: Ras signaling pathway	21	7.55	4.61E-06	3.29	3.18E-05
hsa04010: MAPK signaling pathway	21	7.55	2.79E-05	2.92	1.75E-04
hsa04310: Wnt signaling pathway	13	4.68	4.70E-04	3.34	2.26E-03
hsa04660: T cell receptor signaling pathway	11	3.96	5.89E-04	3.78	2.77E-03
hsa04370: VEGF signaling pathway	8	2.88	1.49E-03	4.65	5.71E-03
hsa04210: Apoptosis	8	2.88	1.64E-03	4.57	6.17E-03

**Table 3:** Significantly represented Biocarta molecular pathways in the Mel38-regulated mRNA gene set.

Biocarta Pathway	Count	Percent of gene set	P-Value	Fold Enrichment	Adjusted P-value (Benjamini)
h_g1Pathway: Cell Cycle: G1/S Check Point	10	3.60	8.61E-05	4.92	0.018
h_cellcyclePathway: Cyclins and Cell Cycle Regulation	9	3.24	1.32E-04	5.32	0.014
h_raccycdPathway: Influence of Ras and Rho proteins on G1 to S Transition	9	3.24	2.41E-04	4.92	0.017
h_p53Pathway: p53 Signaling Pathway	7	2.52	5.35E-04	6.082	0.028
h_rbPathway: RB Tumor Suppressor/Checkpoint Signaling in response to DNA damage	6	2.16	0.0010	6.81	0.043

**Fig 4:**
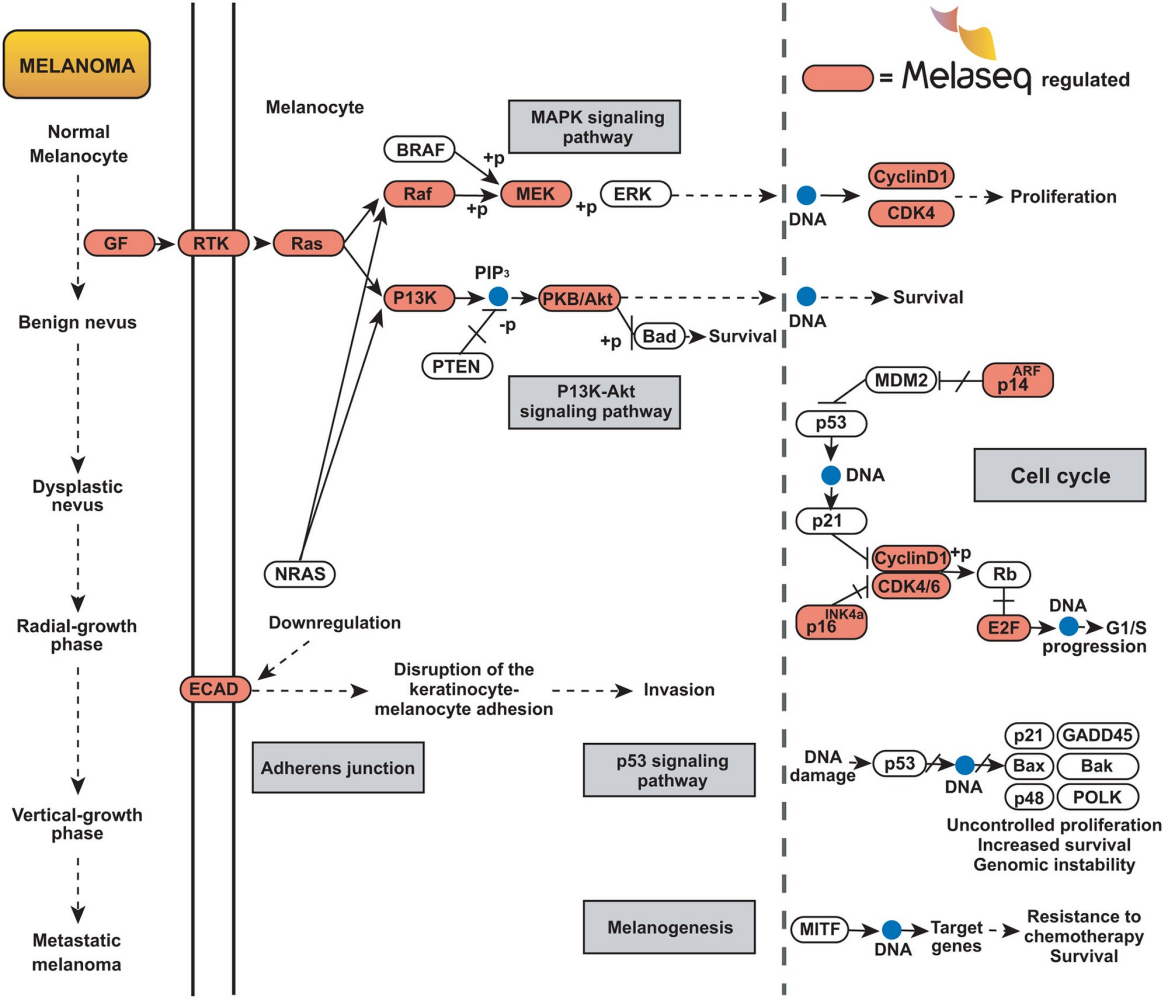
Melanoma KEGG molecular pathway (KEGG ID: hsa05218). Red circles indicate mRNAs (protein coding) genes known to be regulated by one or more of the Mel38 microRNAs. P-value of Mel38-mRNA vs KEGG melanoma pathway overlap: P = 1.3 x 10^−11^.

Notably, the KEGG database’s ‘Melanoma’ pathway (hsa05218) was identified as being significantly represented in the list of 278 Mel38 targets (P = 1.33 x 10–11) and is shown in detail in Fig 4. This pathway describes the molecular events known to occur during the transition from normal melanocyte to metastatic melanoma, including oncogenic NRAS and BRAF mutations, which activate Raf-MEK-ERK and PI3K-Akt, leading to increased cellular proliferation and cell survival(ref). The 19 individual mRNAs that are targeted and/or regulated by the Mel38 signature are highlighted.

Additional characterisation of the 278 Mel-38 regulated genes was performed using gene ontology analysis via the PANTHER classification system[21]. Significantly enriched biological processes are shown in Table 4. This analysis again describes a gene set representing important events in melanomagenesis, including cellular growth, angiogenesis, regulation of apoptosis/survival and BRAF-MAPk signalling.

**Table 4:** Significantly enriched PANTHER gene ontologies identified in the Mel38-regulated mRNA gene set.

PANTHER GO-Slim Biological Process	No. genes	Fold Enrichment	P value	FDR
Growth	2	18.39	7.51E-03	4.70E-02
Negative regulation of apoptotic process	17	12.63	2.83E-13	3.45E-11
Angiogenesis	3	12.26	2.65E-03	1.85E-02
Glycolysis	3	7.88	8.13E-03	4.96E-02
Cell proliferation	6	6.59	4.42E-04	4.32E-03
MAPK cascade	22	4.76	4.36E-09	1.06E-07
Transmembrane receptor protein tyrosine kinase signalling pathway	9	4.39	3.19E-04	3.24E-03
Regulation of phosphate metabolic process	31	4.25	3.89E-11	1.19E-09
Anatomical structure morphogenesis	8	4.17	9.25E-04	7.28E-03
Regulation of catalytic activity	20	4.10	2.25E-07	3.05E-06
Mitosis	12	3.82	1.16E-04	1.23E-03
Cell differentiation	26	3.49	7.13E-08	1.16E-06
Response to endogenous stimulus	9	2.72	7.18E-03	4.61E-02
Regulation of transcription from RNA polymerase II promoter	19	2.34	9.26E-04	7.06E-03
Response to stress	19	2.14	2.95E-03	1.94E-02

#### Protein-coding genes regulated by Mel38 are predictive of melanoma-specific survival

**Fig 5:**
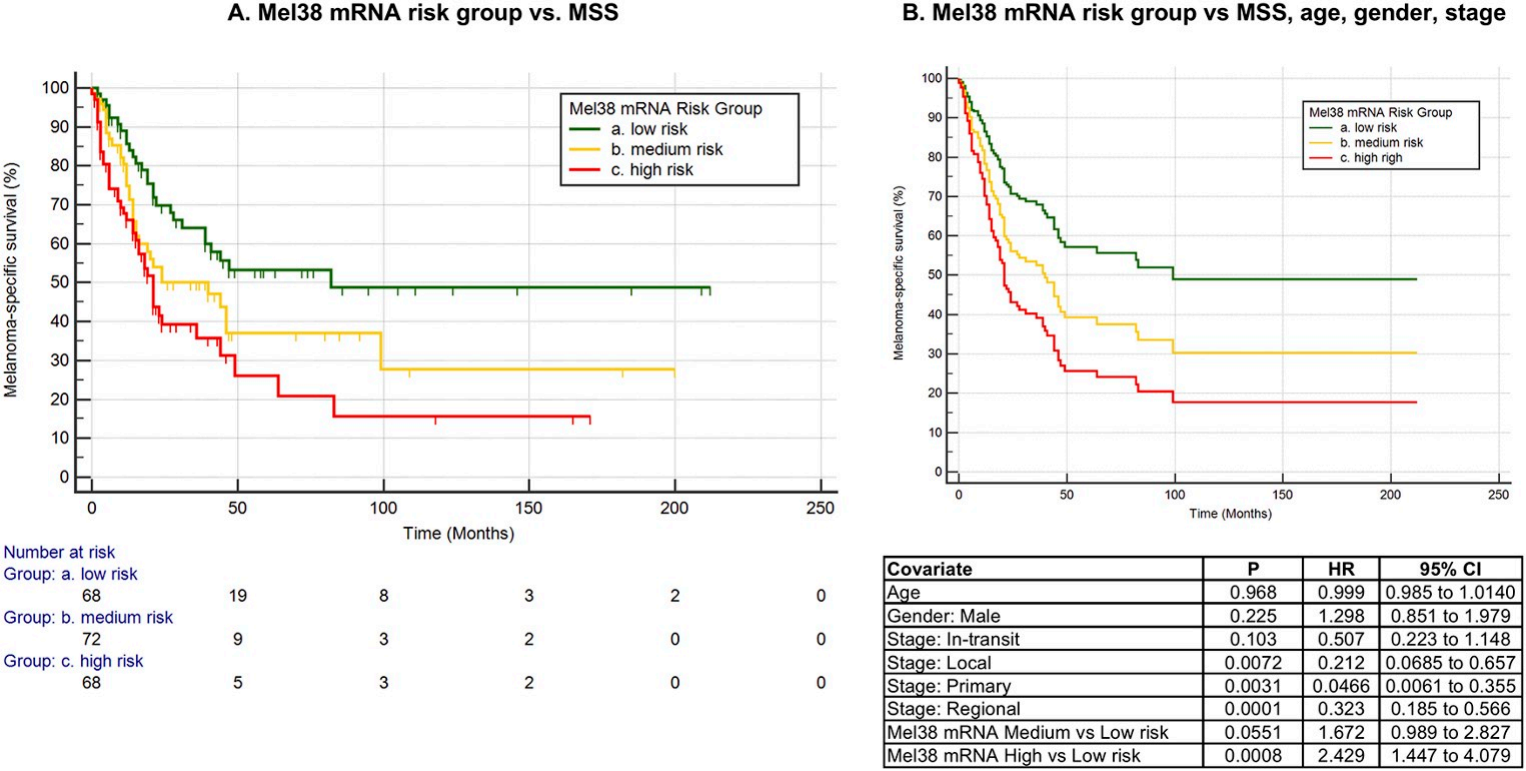
Prognostic stratification of 191 melanoma patients according to Mel38-mRNA expression profiles vs. melanoma-specific-survival (A) Kaplan Meier analysis. Log rank test P = 0.0028 (B) Multivariate cox proportional-hazards regression subgroup plot. Survival differences between the mRNA high vs low risk group is statistically significant (P = 0.0008, Hazard ratio (HR): 2.49), while the difference between the medium and low risk group approached significance (P = 0.055, HR: 1.62), when adjusted for age, gender and stage. Disease stage results are vs the ‘general’ metastasis category.

To examine the prognostic association of the Mel38-regulated mRNA’s, data from the population-based series GSE65904 were again analysed in relation to melanoma specific survival. The 278 mRNA expression levels generated from Illumina BeadChip profiling of 214 unique, fresh-frozen melanoma biopsies were used to train a weighted principal component model, as previously described. Kaplan Meier analysis of the two risk groups, based on 50^th^ percentile prognostic risk score stratification, showed a statistically significant difference in melanoma-specific survival (Fig 5A, log rank test P = 0.0025, hazard ratio = 1.80 (95% CI: 1.22 to 2.67).

Multivariate cox proportional hazards regression incorporating age, gender, melanoma/metastasis type (primary, local, in-transit, regional or general) and genomic risk group was also performed (Fig 5B). When adjusted for these other potentially prognostic variables, the Mel38-regulated mRNA genomic risk groups retained their prognostic significance (High vs. low risk: P = 0.0008, HR: 2.43, 95% CI: 1.45 to 4.079, medium vs low risk: P = 0.055, HR: 1.67, 95% CI: 0.99 to 2.82). As shown in the table of covariates in Fig 5b, primary melanoma vs general metastasis, as well as local/regional metastasis vs general metastases categories were also significant (all P<0.01). These data support the hypotesis that the genomic processes regulated by the Mel38 microRNAs are significantly associated with patient survival, independent to other commonly-used prognostic clinical variables.

## Discussion

It is well established that microRNAs, either circulating in the blood stream or in skin cells and microenvironments, have key roles in melanoma development and progression. Their molecular stability and tissue/disease-state specificity make them ideal biomarker candidates. Despite this, none of the published genomic signatures have been successfully translated into clinical practice. In this study, a recently discovered circulating microRNA signature of melanoma was applied to multiple independent melanoma datasets to demonstrate its utility to assist in the diagnosis of melanoma using FFPE tissue, which is currently considered to be the gold standard method. The relevance of the Mel38 signature to melanoma progression was additionally demonstrated by describing the relationship of the 38 microRNAs to stage III patient outcome and the downstream mRNAs to patient outcome in a population-based patient series.

To our knowledge, this is the first time a novel microRNA signature of melanoma has been successfully applied to archival datasets, generated using multiple genomic profiling technologies. This is an important step in assay validation, in order to assess the robustness of the algorithm in the presence of biological and technical variation. Translation of a genomic signature identified in human plasma, to solid normal and tumour tissue, also supports the hypothesis that the majority of the 38 unique circulating microRNAs discovered in our original study originate from skin cells.

Translation of the 38 microRNAs to protein-coding messenger RNAs resulted in a 278 gene set significantly enriched for molecular pathways with key roles in melanoma development and progression. The most notable pathway identified was the melanocyte to melanoma KEGG pathway, which contains the MAPK signalling sub-pathway; a fundamental step sequence of steps that results in abnormal cellular proliferation. This further supporting the hypothesis that the plasma-detectable microRNA gene set is instrumental in regulating cellular and molecular functions that are specific to melanoma, particularly those involved early stage disease.

A second prognostic model trained on a Mel38-regulated mRNA dataset showed also showed statistically significant differences in patient outcome. The two prognostic analyses in this study suggest the signature may be useful in identifying those patients with more aggressive disease who may benefit from additional therapy or clinical trial enrolment, in addition to its diagnostic and post-treatment monitoring potential.

An important caveat to the results presented in this study is the fact that not all microRNAs present in the Mel38 signature were present on the genomic platforms used to generate the microRNA datasets analysed. This is due to Mel38 being developed using the Nanostring Human MicroRNA v3 codeset, which is based on V21 release of MirBase plus additional clinically-relevant microRNAs identified by the manufacturer [22]. The two previously published microRNA datasets analysed herein were based on MirBase V16 and represent a smaller view of the known microRNAome.

We anticipate that future studies of Mel38 in FFPE tissue and other biofluids will be performed using the full set of 38 microRNAs. This may lead to differences in the diagnostic and prognostic characteristics of signature compared to those reported in this study. It will also be necessary to reassess the prognostic significance of the Mel38 in relation to the updated AJCC 8^th^ edition staging guidelines, which particularly impact on outcome predictions for patients with stage III melanoma [23].

## Conclusion

Despite differences in sample preparation, RNA isolation, detection chemistry and gene set overlap, we have demonstrated that the Mel38 microRNA signature exhibits robust disease-state specificity in solid tissue. This supports the melanoma cell of origin hypothesis for the plasma-identified signature.

Furthermore, in a series of patients with stage III disease, the signature is able to stratify individuals into two risk groups with statistically significant difference in 5-year melanoma-specific survival.

MicroRNA to mRNA mapping revealed that the protein coding genes regulated by Mel38 are involved in multiple oncogenic pathways. These mRNAs also have statistically significant association with patient outcome in univariate and multivariate analyses.

Additional studies are planned to further investigate these observations, using the Nanostring microRNA panel on which the signature was discovered. This will enable future assessments of the signatures diagnostic and prognostic potential to be made using the full 38-microRNA set and with more consistent specimen processing techniques.

Combined with our previous studies, these data show that Mel38 is a robust microRNA signature of cutaneous melanoma. The signature can be used to generate personalised diagnostic or prognostic information about a patient, using plasma or solid tissue. Translation of this signature into a clinically available assay has the potential to improve melanoma diagnostic accuracy and help healthcare professionals identify the disease in its earliest and most curable stage.

## References

[1] Welch HG, Woloshin S, Schwartz LM. Skin biopsy rates and incidence of melanoma: population based ecological study. BMJ. 2005;331(7515):481 Epub 2005/08/04. 10.1136/bmj.38516.649537.E0 . 16081427 PMC1199022

[2] Australian Cancer Incidence and Mortality (ACIM) books. In: Welfare AIoHa, editor. 03 Feb 2017 ed: Australian Government; 2017.

[3] Bray F, Ferlay J, Soerjomataram I, Siegel RL, Torre LA, Jemal A. Global cancer statistics 2018: GLOBOCAN estimates of incidence and mortality worldwide for 36 cancers in 185 countries. CA Cancer J Clin. 2018;68(6):394–424. Epub 2018/09/13. 10.3322/caac.21492 . 30207593

[4] Elmore JG, Barnhill RL, Elder DE, Longton GM, Pepe MS, Reisch LM, et al Pathologists’ diagnosis of invasive melanoma and melanocytic proliferations: observer accuracy and reproducibility study. BMJ. 2017;357. 10.1136/bmj.j2813 PMC5485913 28659278

[5] Corona R, Mele A, Amini M, De Rosa G, Coppola G, Piccardi P, et al Interobserver variability on the histopathologic diagnosis of cutaneous melanoma and other pigmented skin lesions. J Clin Oncol. 1996;14(4):1218–23. 10.1200/JCO.1996.14.4.1218 . 8648377

[6] Gerami P, Busam K, Cochran A, Cook MG, Duncan LM, Elder DE, et al Histomorphologic assessment and interobserver diagnostic reproducibility of atypical spitzoid melanocytic neoplasms with long-term follow-up. Am J Surg Pathol. 2014;38(7):934–40. 10.1097/PAS.0000000000000198 . 24618612

[7] Sidhu S, Bodger O, Williams N, Roberts DL. The number of benign moles excised for each malignant melanoma: the number needed to treat. Clin Exp Dermatol. 2012;37(1):6–9. Epub 2011/10/07. 10.1111/j.1365-2230.2011.04148.x . 21981313

[8] Baade PD, Youl PH, Janda M, Whiteman DC, Del Mar CB, Aitken JF. Factors associated with the number of lesions excised for each skin cancer: a study of primary care physicians in Queensland, Australia. Arch Dermatol. 2008;144(11):1468–76. 10.1001/archderm.144.11.1468 . 19015421

[9] Ferlay J, Soerjomataram I, M E. Cancer Incidence and Mortality Worldwide: IARC CancerBase Lyon, France2018 [cited 2018 September]. The Global Cancer Observatory (GCO) is an interactive web-based platform presenting global cancer statistics to inform cancer control and research.]. http://gco.iarc.fr/.

[10] Wahie S, Lawrence CM. Wound complications following diagnostic skin biopsies in dermatology inpatients. Arch Dermatol. 2007;143(10):1267–71. 10.1001/archderm.143.10.1267 . 17938340

[11] Van Laar R, Lincoln M, Van Laar B. Development and validation of a plasma-based melanoma biomarker suitable for clinical use. Br J Cancer. 2018 Epub 2018/01/23. 10.1038/bjc.2017.477 . 29360813 PMC5886119

[12] van Laar RK, Lincoln MT, van Laar BJ. A plasma microRNA biomarker of melanoma as a personalised assessment of treatment response. Melanoma Res. 2019;29(1):19–22. Epub 2018/10/16. 10.1097/CMR.0000000000000492 . 30320629

[13] Edgar R, Domrachev M, Lash A. Gene Expression Omnibus: NCBI gene expression and hybridization array data repository. Nucl Acid Res. 2002;30:207–10. 10.1093/nar/30.1.207 PMC99122 11752295

[14] Xu Y, Brenn T, Brown ER, Doherty V, Melton DW. Differential expression of microRNAs during melanoma progression: miR-200c, miR-205 and miR-211 are downregulated in melanoma and act as tumour suppressors. Br J Cancer. 2012;106(3):553–61. Epub 2012/01/05. 10.1038/bjc.2011.568 . 22223089 PMC3273359

[15] Jayawardana K, Schramm SJ, Haydu L, Thompson JF, Scolyer RA, Mann GJ, et al Determination of prognosis in metastatic melanoma through integration of clinico-pathologic, mutation, mRNA, microRNA, and protein information. Int J Cancer. 2015;136(4):863–74. 10.1002/ijc.29047 . 24975271

[16] Cirenajwis H, Ekedahl H, Lauss M, Harbst K, Carneiro A, Enoksson J, et al Molecular stratification of metastatic melanoma using gene expression profiling: Prediction of survival outcome and benefit from molecular targeted therapy. Oncotarget. 2015;6(14):12297–309. 10.18632/oncotarget.3655 . 25909218 PMC4494939

[17] Bair E, Tibshirani R. Semi-supervised methods to predict patient survival from gene expression data. PLoS Biol. 2004;2(4):E108 Epub 2004/04/20. 10.1371/journal.pbio.0020108 . 15094809 PMC387275

[18] Schoonjans F, Zalata A, Depuydt CE, Comhaire FH. MedCalc: a new computer program for medical statistics. Comput Methods Programs Biomed. 1995;48(3):257–62. . 8925653 10.1016/0169-2607(95)01703-8

[19] Huang DW, Sherman BT, Lempicki RA. Systematic and integrative analysis of large gene lists using DAVID bioinformatics resources. Nat Protocols. 2008;4(1):44–57. 10.1038/nprot.2008.211 19131956

[20] Chou CH, Shrestha S, Yang CD, Chang NW, Lin YL, Liao KW, et al miRTarBase update 2018: a resource for experimentally validated microRNA-target interactions. Nucleic Acids Res. 2018;46(D1):D296–D302. 10.1093/nar/gkx1067 . 29126174 PMC5753222

[21] Mi H, Huang X, Muruganujan A, Tang H, Mills C, Kang D, et al PANTHER version 11: expanded annotation data from Gene Ontology and Reactome pathways, and data analysis tool enhancements. Nucleic Acids Res. 2017;45(D1):D183–D9. Epub 2016/11/29. 10.1093/nar/gkw1138 . 27899595 PMC5210595

[22] Sundarbose K, Kartha RV, Subramanian S. MicroRNAs as Biomarkers in Cancer. Diagnostics (Basel). 2013;3(1):84–104. Epub 2013/01/16. 10.3390/diagnostics3010084 . 26835669 PMC4665585

[23] Gershenwald JE, Scolyer RA, Hess KR, Sondak VK, Long GV, Ross MI, et al Melanoma staging: Evidence-based changes in the American Joint Committee on Cancer eighth edition cancer staging manual. CA Cancer J Clin. 2017;67(6):472–92. Epub 2017/10/13. 10.3322/caac.21409 . 29028110 PMC5978683

